# Identification of optimal cutoffs for obesity and lipid-related indices in screening activities of daily living disability in Chinese middle-aged and elderly adults

**DOI:** 10.3389/fnut.2025.1599851

**Published:** 2025-07-09

**Authors:** Jian Jiang, Shun Liu, Nan Wei, Zhifeng Lin, Yiming Hu, Xiaoqi Chen, Ling Yao, Zhiying Yao, Qingquan Chen

**Affiliations:** ^1^Fuzhou University Affiliated Provincial Hospital; Shengli Clinical Medical College of Fujian Medical University, Fujian Provincial Key Laboratory of Emergency Medicine, Fujian Emergency Medical Center, Fuzhou, China; ^2^Fujian Medical University, Fuzhou, China; ^3^Xiangya Hospital, Central South University, Changsha, China

**Keywords:** activities of daily living, obesity and lipid-related measurement indices, cross-sectional studies, CHARLS database, receiver operating characteristic curve

## Abstract

**Objective:**

The aim of this study was to investigate the efficacy of 13 obesity- and lipid-related indices in screening and predicting activities of daily living (ADL) disability, as well as to determine the optimal cutoffs for these indices in the middle-aged and elderly population in China.

**Methods:**

This study was based on cross-sectional data from the China Health and Retirement Longitudinal Study (CHARLS), which included 4,784 participants aged 45 years and older. Thirteen obesity- and lipid-related indices, including body mass index (BMI), waist circumference (WC), the waist–height ratio (WHtR), the visceral adiposity index (VAI), the body shape index (ABSI), the body roundness index (BRI), the lipid accumulation product (LAP), the conicity index (CI), the Chinese visceral adiposity index (CVAI), the triglyceride–glucose (TyG) index and its correlation index (TyG–BMI, TyG–WC, TyG–WHtR), were analyzed. Unadjusted and adjusted correlations between these indices and ADL disability were assessed via logistic regression analyses, and the area under the curve (AUC) of each index as a predictor of ADL ability was calculated via receiver operating characteristic (ROC) curves to compare the predictive efficacy and determine the optimal cutoff value.

**Results:**

After adjustment for confounders, all 13 obesity- and lipid-related indices were independently associated with the risk of ADL disability (*p* < 0.05). All 13 indices had predictive value for ADL disability according to the ROC analysis (all AUCs > 0.7). The LAP index exhibited the highest predictive efficacy in men (AUC = 0.793, 95% CI: 0.704–0.882, and optimal cutoff = 35.669), and BMI was optimal in women (AUC = 0.721, 95% CI: 0.678–0.765, and optimal cutoff = 26.142). The TyG-BMI performed well in both sexes (men AUC = 0.790, female AUC = 0.720). The risk of ADL disability increased significantly with each unit increase in obesity and lipid indices.

**Conclusion:**

All 13 obesity- and lipid-related indices were effective predictors of ADL disability risk in Chinese middle-aged and older adults. LAP and BMI emerged as the best predictors of ADL disability in men and women, respectively. These indices can serve as simple screening tools to identify the risk of ADL disability and facilitate early intervention.

## 1 Introduction

Population aging has emerged as a critical demographic challenge confronting nations worldwide, including China. From 2025 to 2,100, the proportion of the elderly population is projected to continue increasing, with more than 30% of the population aged 65 years and over by 2,100. During this period, the dependency ratio of the elderly population is expected to reach 4:1 (1). The exploding elderly population has led to a surge in the prevalence and incidence of age-related diseases, posing significant public health challenges (2). Moreover, age-related health problems are likely to result in disability in activities of daily living (ADL) (3). ADL refers to activities oriented toward taking care of one’s own body, which are fundamental to living in a social world. These activities enable basic survival and well-being, including bathing, toileting, dressing, and eating, among others (4, 5). It is estimated that more than 1 billion people worldwide suffer from one or more forms of disability (6). Reduced ADL capacity negatively affects the physical and mental health of middle-aged and older adults, increases the risk of unintentional injuries, and may even be associated with a greater risk of mortality (7). Recent studies in China have shown that the prevalence of ADL disability among older adults is 41.0%, and this number is expected to continue to increase as the population ages (8). This trend places a heavy burden on individuals, caregivers, and the healthcare system. Therefore, early identification of people at risk for ADL disability is critical for both prevention and intervention efforts.

Moreover, the high prevalence of obesity has become a major global health problem, increasing at an alarming rate in recent decades (9). Obesity has been identified as a significant risk factor for dyslipidemia, with the underlying mechanism potentially related to insulin resistance (IR) (10). In the context of IR, the clearance of triglyceride (TG)-rich lipoproteins from the blood is impaired, leading to elevated TG levels and ultimately lower high-density lipoprotein cholesterol (HDL-C) levels (11, 12). In addition, obesity is thought to significantly increase the risk of adverse health outcomes in middle-aged and older populations, including a variety of comorbidities such as hypertension and diabetes, as well as functional impairments, such as mobility problems (13, 14). Annemarie Koster et al. demonstrated that obese older adults are at greater risk for activity limitations than nonobese individuals are and that the effects of obesity appear to partially overwhelm the effects of other lifestyle factors (15). Another study in a Chinese middle-aged and elderly population showed that power-reduced abdominal obesity is associated with functional disability, i.e., difficulty performing basic activities of daily living (BADL) and instrumental activities of daily living (IADL) (16).

Waist circumference (WC) and body mass index (BMI) are the most commonly used measures of obesity in previous studies. Although they have been widely used in many studies to explore the associations between obesity and certain diseases (diabetes, metabolic syndrome (MS), and depressive symptoms), these metrics have certain shortcomings (17, 18). In muscular individuals, BMI fails to differentiate between muscle mass and fat mass, leading to erroneous assessment results (9, 19). Similarly, the strong correlation between WC and BMI complicates the ability to distinguish their respective contributions as independent epidemiologic risk factors (20). Given these limitations, the representativeness of the results could be enhanced by introducing additional anthropometric indices to compare the predictive power of obesity and lipid-related indices comprehensively.

In view of this, the current study employed 13 obesity- and lipid-related indices to assess body fat accumulation and lipid metabolite status, utilizing data from the China Health and Retirement Longitudinal Study (CHARLS). These indices included WC, BMI, the waist–height ratio (WHtR), the visceral adiposity index (VAI), the body shape index (ABSI), the body roundness index (BRI), the lipid accumulation product (LAP), the conicity index (CI), the Chinese visceral adiposity index (CVAI), the triglyceride–glucose (TyG) index and its correlation index (TyG–BMI, TyG–WC, TyG–WHtR). The primary objectives were to investigate the screening and predictive efficacy of these obesity- and lipid-related indices for the risk of ADL disability in middle-aged and older adults and to determine the optimal predictive cutoff values to provide a basis for the prevention and treatment of ADL disability.

## 2 Methods

### 2.1 Study design and setting

The data for this study were sourced from CHARLS, a nationally representative cohort study of middle-aged and older adults aged 45–101 years in China. All the data are publicly available as microdata at http://charls.pku.edu.cn/index/zh-cn.html without any direct human interaction. Informed consent was obtained from all participants prior to data collection, and the study was approved by the Ethics Committee of the China Center for Economic Research at Peking University.

### 2.2 Participants

**Figure 1:**
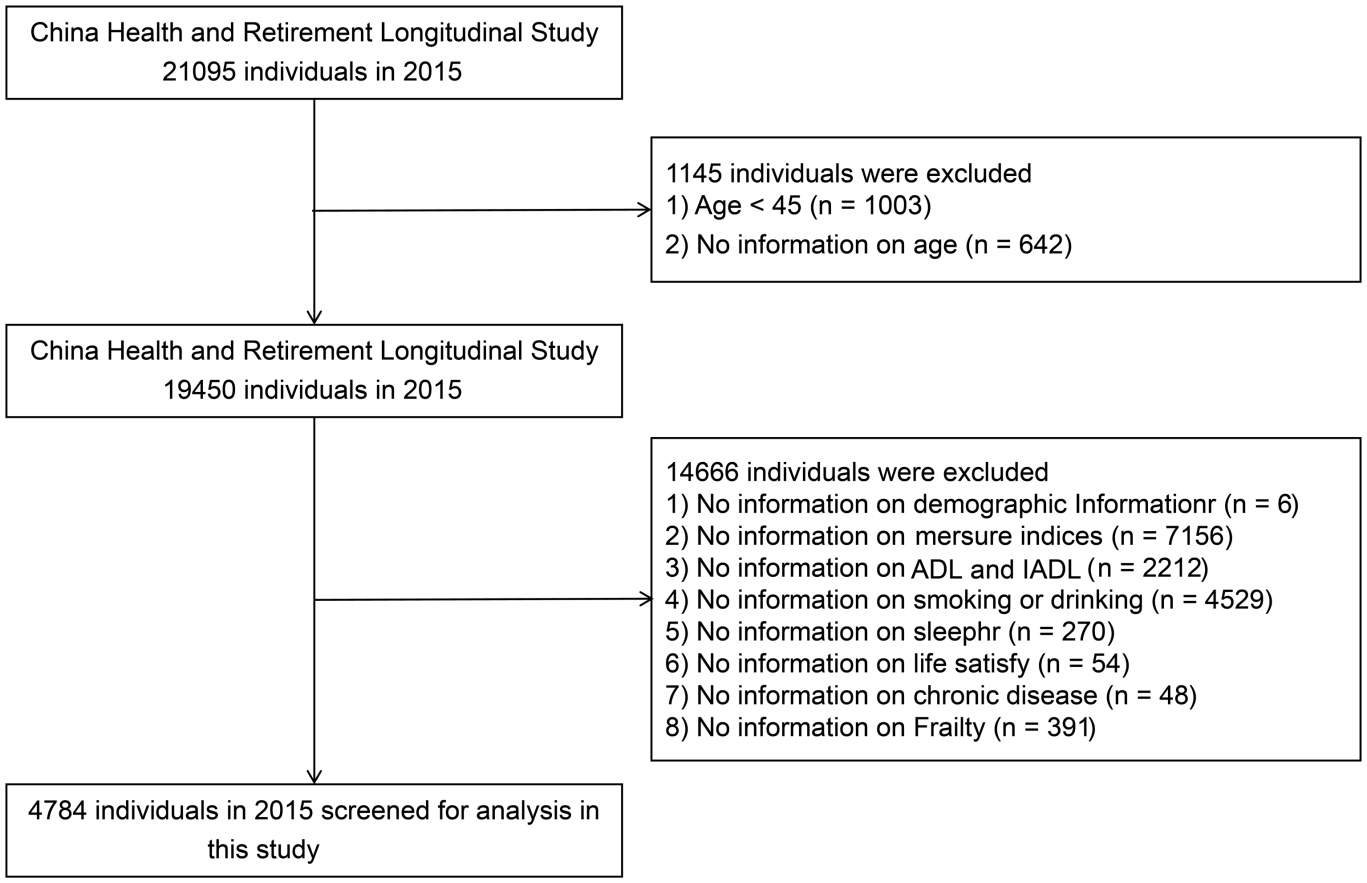
Flowchart of data screening for this study.

Data from Wave 3 of CHARLS were used in this study. We excluded individuals who met any of the following criteria at baseline (see Figure 1): (1) missing data on ADLs; (2) age under 45 years; and (3) missing data on age/sex/education level/marital status/current residence/smoking/alcohol consumption/frailty status/human body measurement index/sleeping time/life satisfaction/hypertension/high blood pressure/high blood lipid/high blood glucose. After these cases were excluded, a total of 4,784 individuals aged 45 years and above were included in the analysis. Among them, 811 (17.0%) were males, and 3,973 (83.0%) were females. Considering the imbalance between the male and female included populations, we conducted a gender-stratified study in the subsequent analysis.

### 2.3 Disability in activities of daily living

Activities of daily living (ADL) disability are defined as being dependent on or needing help from others in at least one of the six basic skills normally required to meet our basic physical needs. These skills include grooming and personal hygiene, dressing, toileting, transferring and walking, and eating. For each ADL item, participants were asked to choose one of the following four responses: (1) No, I do not have any difficulty; (2) I have difficulty, but I can still do it; (3) Yes, I have difficulty and need help; and (4) I cannot do it. A score of 3 or 4 indicates a disability in this ADL program.

### 2.4 Anthropometric indices

Anthropometric measurements were conducted by certified medical personnel. Weight and height were estimated to the nearest 0.1 kg and 0.5 cm, respectively, via standard medical equipment. After exhalation, the abdominal circumference was measured between the iliac crest and the lower edge of the ribs on both sides. BMI was measured as weight (kg)/height^2^ (m^2^). WHtR is defined as WC (cm)/height (cm). The VAI, CVAI, LAP, and TyG index require TG and HDL-C to be obtained via blood sample collection for calculation. The TyG index is the result of a computation using TG and glucose. Except for WC, the remaining anthropometric indices were calculated via the following equations.

$\mathrm{BMI}=\frac{\text{Weight}\;}{{\text{Height}}^{2}\;}$

$\text{WHtR}=\frac{\mathrm{WC}}{\text{Height}}$

$\begin{array}{l} \text{Males}:\\ \mathrm{VAI}=\frac{\mathrm{WC}}{39.68+(1.88\times \mathrm{BMI})}\times \frac{\mathrm{TG}}{1.03}\times \frac{1.31}{\mathrm{HDL}}\\ \text{Females}:\\ \mathrm{VAI}=\frac{\mathrm{WC}}{36.58+(1.89\times \mathrm{BMI})}\times \frac{\mathrm{TG}}{0.81}\times \frac{1.52}{\mathrm{HDL}} \end{array}$

$\text{ABSI}=\frac{\mathrm{WC}}{{\text{Height}}^{1/2}\times \mathrm{BM}I^{2/3}}$

$\mathrm{BRI}=364.2‐365.5\sqrt{1‐(\frac{{\left(\mathrm{WC}(m)/(2,\pi )\right)}^{2}}{{\left(0.5\times H\text{eight}(m)\right)}^{2}})}\;$

$\begin{array}{l} \text{Males}:\\ \mathrm{LAP}=|\mathrm{WC}\;(\mathrm{cm})‐65|\times \mathrm{TG}\;(\text{mmol}/L)\\ \text{Females}:\\ \mathrm{LAP}=|\mathrm{WC}\;(\mathrm{cm})‐58|\times \mathrm{TG}\;(\text{mmol}/L) \end{array}$

$\mathrm{CI}=\frac{\mathrm{WC}(m)}{0.019\sqrt{\frac{\text{Weight}\;(\mathrm{kg})}{\text{Height}\;(m)}}}$

$\begin{array}{l} \text{Males}:\\ \begin{array}{l} \text{CVAI}=-267.93+0.68\times \mathrm{Age}+0.03\times \mathrm{BMI}+4.00\times \\ \mathrm{WC}+22.00\times \log_{10}(\mathrm{TG})-16.32\times \mathrm{HDL}-C \end{array}\\ \text{Females}:\\ \begin{array}{l} \text{CVAI}=-187.32+1.71\times \mathrm{Age}+4.32\times \mathrm{BMI}+1.12\times \mathrm{WC}+\\ 39.76\times \log_{10}(\mathrm{TG})-11.66\times \mathrm{HDL}-C \end{array} \end{array}\mathrm{TyG}\;\text{Index}=\ln\;[(\mathrm{TG}\;(\mathrm{mg}/\mathrm{dL})\times \text{Glucose}\;(\mathrm{mg}/\mathrm{dL})/2]$

TyG‐BMI=TyG×BMI

TyG‐WC=TyG×WC

TyG‐WHtR=TyG×WHtR

### 2.5 Covariates

Sociodemographic characteristics, including age, sex (1 = male, 2 = female), education, marital status, current place of residence, current smoking status, alcohol consumption, obesity status, sleep duration, life satisfaction, hypertension, hyperglycemia, and hyperlipidemia, were collected via self-report questionnaires. These categorizations have been widely used in our previous studies.

a) Age was categorized as 45–50/51–60/61–70/≥ 71 years or older.
b) Education was categorized as no formal education/primary school/middle school/high school/high school+.
c) Marital status was categorized as single/married.
d) Current place of residence was categorized as urban/rural.
e) Current smoking status was categorized as nonsmoker/smoker.
f) Alcohol consumption was categorized as never drinking/alcohol consumption.
g) Obesity was categorized as currently obese/not obese.
h) Life satisfaction was categorized as dissatisfied/satisfied.
i) Frailty was categorized as having or not having frailty status.
j) IADL was categorized as having or not having IADL.
k) Sleep duration was categorized as night bedtime and midday bedtime.

i) Hypertension/hyperglycemia/hyperlipidemia were categorized as present and absent, respectively.

### 2.6 Statistical analysis

All the statistical analyses were performed via R software (version 4.2.3). Categorical variables are expressed as frequencies and percentages. Comparisons of dichotomous or multicategorical variables were made via the chi-square test and one-way ANOVA. Continuous variables are expressed as the means and standard deviations. Unadjusted and adjusted correlations between obesity and lipid-related indicators and ADLs were assessed via binary logistic regression analysis. Odds ratios (ORs) and 95% confidence intervals (95% CIs) of obesity and lipid-related indicators associated with ADLs were calculated after adjusting for age, educational attainment, marital status, current place of residence, smoking, alcohol consumption, and chronic diseases. The area under the curve (AUC) for each indicator as a predictor of ADLs was calculated via the receiver operating characteristic (ROC) curve after accounting for the effects of age, education, marital status, and place of residence. The indicator with the highest AUC was considered the most accurate indicator, and the closer the AUC was to 1, the more accurate the prediction was. Optimal cutoffs were calculated for obesity and lipid-related indicators. Statistical significance was defined as *p* < 0.05.

## 3 Results

Table 1 presents the baseline characteristics of the participants according to the presence or absence of ADL disability. The mean age of the participants was 60.2 ± 9.5 years, with 83.0% being female, 85.5% being married, and 83.2% residing in rural areas. The prevalence of smoking was 8.0%, alcohol consumption was 13.4%, obesity was 53.0%, hypertension was 30.7%, hyperlipidemia was 24.7%, and hyperglycemia was 15.4%. Additionally, 90.2% of the participants reported being satisfied with their life, 12.4% had frailty, and 9.7% had IADL disability. Significant differences were observed between participants with and without ADL disability in terms of age, marital status, place of residence, hypertension, hyperlipidemia, life satisfaction, IADL disability, frailty, night sleep duration, triglyceride (TG) level, and height.

**Table 1:** Baseline characteristics of participants grouped by presence or absence of ADL.

Variables	No ADL (*N* = 4,651)	ADL (*N* = 133)	Total (*N* = 385)	*p*
Age (years)	60.0 ± 9.5	66.0 ± 8.7	60.2 ± 9.5	<0.001
Age, *n* (%)				
45–50	892 (19.2)	9 (6.8)	901 (18.8)	<0.001
51–60	1,570 (33.8)	25 (18.8)	1,595 (33.3)	
61–70	1,493 (32.1)	59 (44.4)	1,552 (32.4)	
≥71	696 (15.0)	40 (30.1)	736 (15.4)	
Marital, *n* (%)				
Married	3,991 (85.8)	101 (75.9)	4,092 (85.5)	0.002
Single	660 (14.2)	32 (24.1)	692 (14.5)	
Education, *n* (%)				
No formal education	422 (9.1)	18 (13.5)	440 (9.2)	0.202
Primary school	506 (10.9)	10 (7.5)	516 (10.8)	
Middle school	162 (3.5)	4 (3.0)	166 (3.5)	
High school	60 (1.3)	0 (0.0)	60 (1.3)	
High school+	3,501 (75.3)	101 (75.9)	3,602 (75.3)	
Residence, *n* (%)				
Rural	3,858 (82.9)	122 (91.7)	3,980 (83.2)	0.011
Urban	793 (17.1)	11 (8.3)	804 (16.8)	
Obesity, *n* (%)				
No	2,195 (47.2)	52 (39.1)	2,247 (47.0)	0.079
Yes	2,456 (52.8)	81 (60.9)	2,537 (53.0)	
Smoking, *n* (%)				
No	4,274 (91.9)	128 (96.2)	4,402 (92.0)	0.097
Yes	377 (8.1)	5 (3.8)	382 (8.0)	
Drinking, *n* (%)				
No	4,024 (86.5)	121 (91.0)	4,145 (86.6)	0.174
Yes	627 (13.5)	12 (9.0)	639 (13.4)	
Life_satisfy, *n* (%)				
Not satisfied	443 (9.5)	25 (18.8)	468 (9.8)	0.001
Satisfied	4,208 (90.5)	108 (81.2)	4,316 (90.2)	
Hypertension, *n* (%)				
No	3,243 (69.7)	72 (54.1)	3,315 (69.3)	<0.001
Yes	1,408 (30.3)	61 (45.9)	1,469 (30.7)	
Dyslipidemia, *n* (%)				
No	3,525 (75.8)	90 (67.7)	3,615 (75.6)	0.041
Yes	1,126 (24.2)	43 (32.3)	1,169 (24.4)	
Diabetes, *n* (%)				
No	3,948 (84.9)	97 (72.9)	4,045 (84.6)	<0.001
Yes	703 (15.1)	36 (27.1)	739 (15.4)	
IADL, *n* (%)				
No	4,256 (91.5)	65 (48.9)	4,321 (90.3)	<0.001
Yes	395 (8.5)	68 (51.1)	463 (9.7)	
Frailty, *n* (%)				
No	4,125 (88.7)	65 (48.9)	4,190 (87.6)	<0.001
Yes	526 (11.3)	68 (51.1)	594 (12.4)	
TG (mg/dl)	147.7 ± 90.2	164.4 ± 93.6	148.1 ± 90.3	0.035
HDL (mg/dl)	51.9 ± 10.8	51.0 ± 14.7	51.9 ± 10.9	0.356
GLU (mg/dl)	104.1 ± 35.8	110.0 ± 49.9	104.2 ± 36.3	0.063
Height (cm)	154.8 ± 7.5	152.3 ± 7.5	154.8 ± 7.5	<0.001
Weight (kg)	59.0 ± 11.4	59.8 ± 15.3	59.0 ± 11.6	0.435
BMI (kg/m2)	24.5 ± 4.0	25.6 ± 5.9	24.6 ± 4.1	0.002
WC (cm)	86.2 ± 13.2	88.9 ± 14.4	86.2 ± 13.2	0.017
WHtR	0.6 ± 0.1	0.6 ± 0.1	0.6 ± 0.1	<0.001
CVAI	107.5 ± 41.0	126.8 ± 48.6	108.0 ± 41.3	<0.001
VAI	5.8 ± 4.7	6.9 ± 5.3	5.8 ± 4.7	0.007
ABSI	0.8 ± 0.1	0.8 ± 0.1	0.8 ± 0.1	0.055
BRI	4.6 ± 1.6	5.3 ± 1.8	4.7 ± 1.6	<0.001
LAP	49.8 ± 40.0	60.2 ± 42.5	50.1 ± 40.1	0.003
CI	7.4 ± 0.9	7.6 ± 1.0	7.4 ± 0.9	0.019
TYG	8.8 ± 0.6	8.9 ± 0.7	8.8 ± 0.6	0.008
TYG_BMI	215.7 ± 42.5	228.6 ± 54.0	216.1 ± 42.9	0.001
TYG_WC	7.6 ± 1.4	7.9 ± 1.5	7.6 ± 1.4	0.003
TYG_WHtR	4.9 ± 0.9	5.2 ± 1.0	4.9 ± 0.9	<0.001
Sleephr_night (hours)	6.2 ± 2.0	5.4 ± 2.1	6.2 ± 2.0	<0.001
Sleephr_lunch (minutes)	36.1 ± 43.8	42.5 ± 48.5	36.3 ± 43.9	0.097

ADL, Activities of Daily Living; IADL, Instrumental Activities of Daily Living; TG, Triglyceride; HDL, High-Density Lipoprotein; GLU, Glucose; WC, waist circumference; BMI, body mass index; WHtR, waist to height ratio; VAI, visceral adiposity index; ABSI, A body shape index; BRI, body roundness index; LAP, lipid accumulation product; CVAI, Chinese visceral adiposity index; CI, conicity index; TyG index, triglyceride-glucose index; TyG-BMI, TyG related to BMI; TyG-WC, TyG related to WC; TyG-WHtR, TyG related to WHtR.

**Figure 2:**
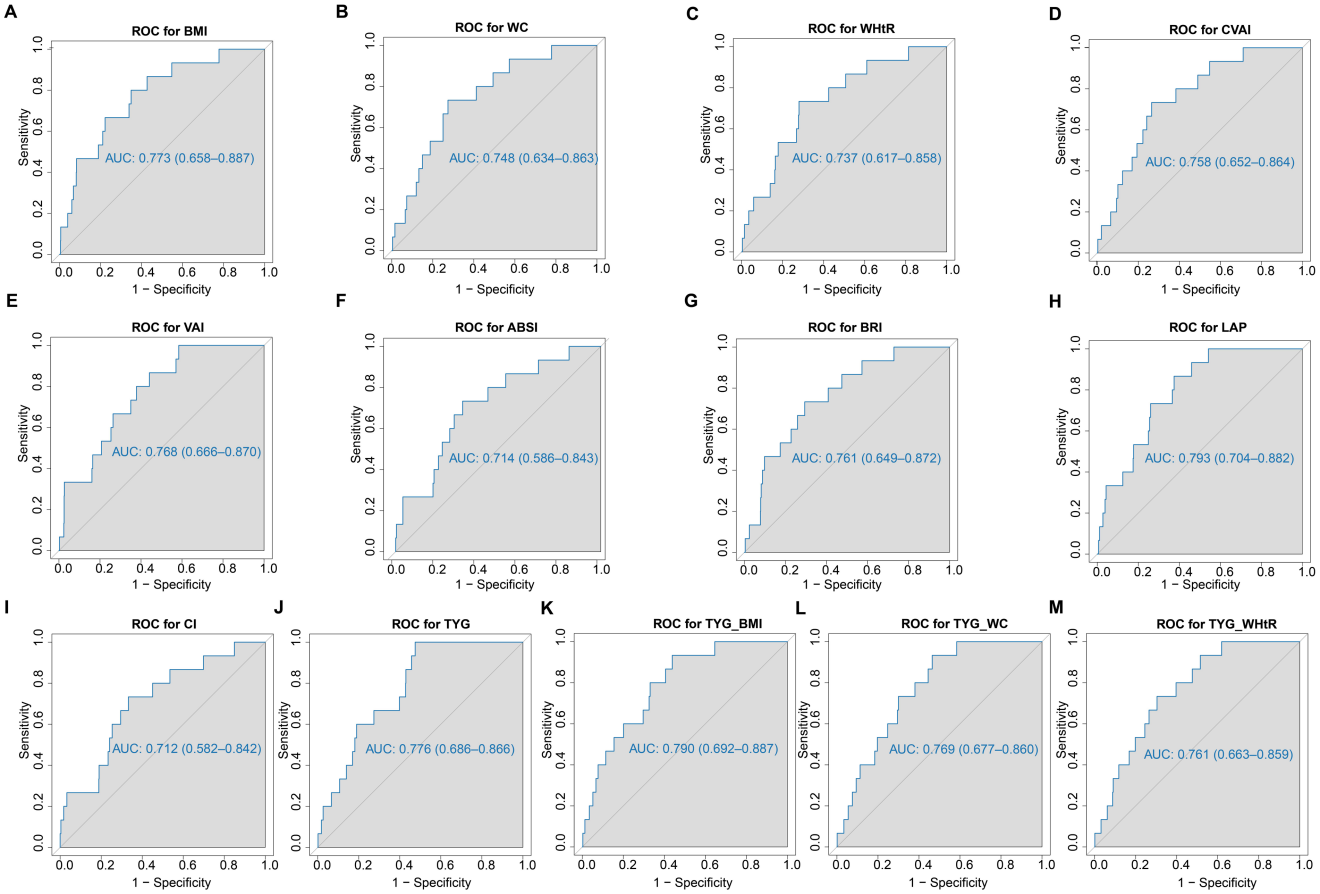
The ROC curves of each indicator in the prediction of ADL risk in males. **(A)** ROC for BMI; **(B)** ROC for WC; **(C)** ROC for WHtR; **(D)** ROC for CVAI; **(E)** ROC for VAI; **(F)** ROC for ABSI; **(G)** ROC for BRI; **(H)** ROC for LAP; **(I)** ROC for CI; **(J)** ROC for TYG; **(K)** ROC for TYG_BMI; **(L)** ROC for TYG_WC; **(M)** ROC for WHtR. WC, waist circumference; BMI, body mass index; WHtR, waist to height ratio; VAI, visceral adiposity index; ABSI, A body shape index; BRI, body roundness index; LAP, lipid accumulation product; CVAI, Chinese visceral adiposity index; CI, conicity index; TyG index, triglyceride-glucose index; TyG-BMI, TyG related to BMI; TyG-WC, TyG related to WC; TyG-WHtR, TyG related to WHtR.

**Figure 3:**
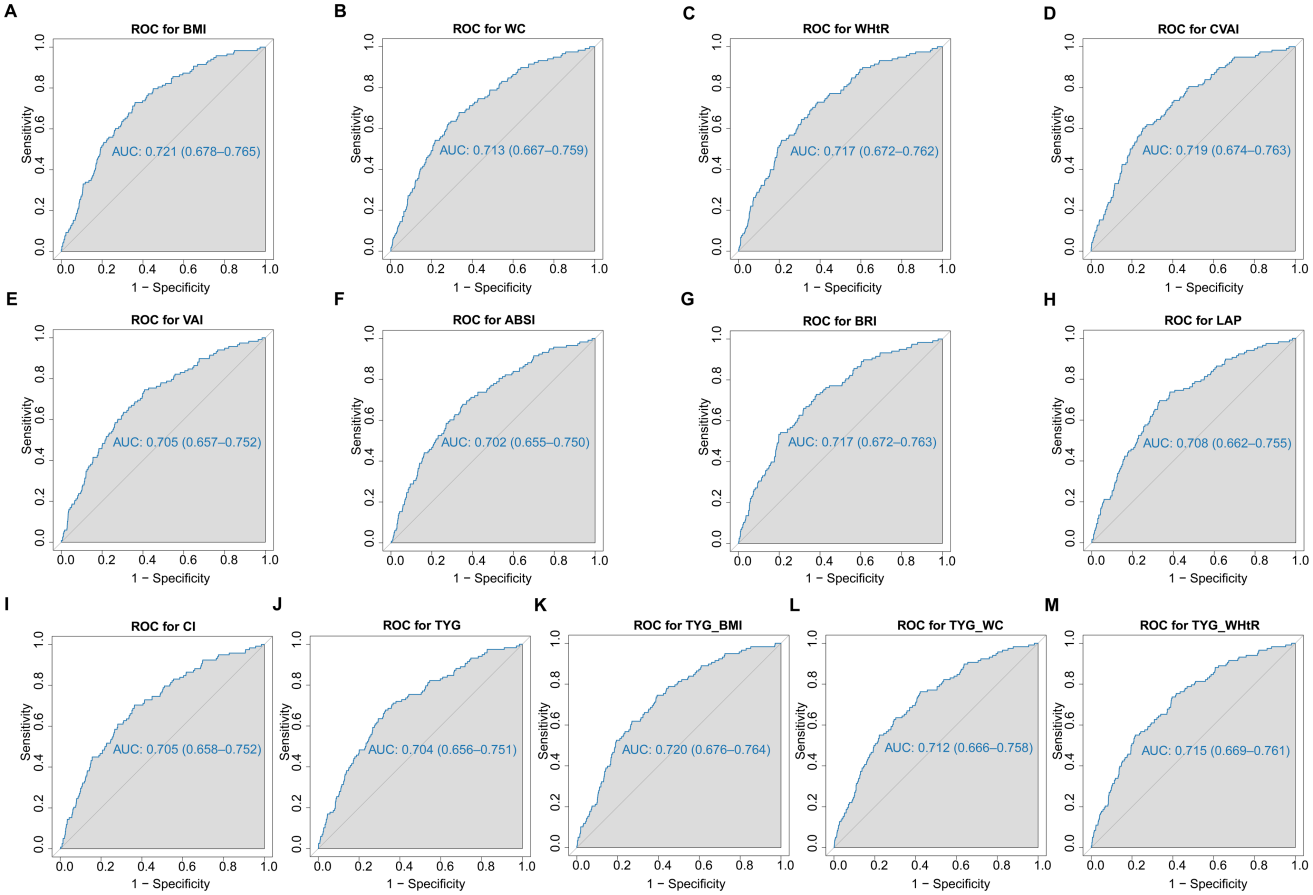
The ROC curves of each indicator in the prediction of ADL risk in females. **(A)** ROC for BMI; **(B)** ROC for WC; **(C)** ROC for WHtR; **(D)** ROC for CVAI; **(E)** ROC for VAI; **(F)** ROC for ABSI; **(G)** ROC for BRI; **(H)** ROC for LAP; **(I)** ROC for CI; **(J)** ROC for TYG; **(K)** ROC for TYG_BMI; **(L)** ROC for TYG_WC; **(M)** ROC for WHtR. WC, waist circumference; BMI, body mass index; WHtR, waist to height ratio; VAI, visceral adiposity index; ABSI, A body shape index; BRI, body roundness index; LAP, lipid accumulation product; CVAI, Chinese visceral adiposity index; CI, conicity index; TyG index, triglyceride-glucose index; TyG-BMI, TyG related to BMI; TyG-WC, TyG related to WC; TyG-WHtR, TyG related to WHtR.

Table 2 shows the AUC, 95% CIs, optimal cutoff values and test statistic *p* values for obesity and lipid-related indices for the prediction of ADL disability by sex. Figures 2, 3 show the ROC curves for each index for predicting the risk of ADL disability in males and females, respectively. The ROC curves for each index for predicting the risk of ADL disability in males and females. As illustrated in Table 2 and Figure 2, in males, the LAP index was the best predictor of ADL disability (AUC = 0.793, 95% CI: 0.704–0.882; optimal cutoff = 35.669). Additionally, the TYG-BMI (AUC = 0.790, 95% CI: 0.692–0.887, and optimal cutoff = 239.694) had a similar predictive value. Furthermore, in women, BMI was the most accurate predictor of ADL disability (AUC = 0.721, 95% CI: 0.678–0.765, and optimal cutoff = 26.142), followed by TYG-BMI (AUC = 0.720, 95% CI: 0.676–0.764, and optimal cutoff = 224.475). All of the above indicators were statistically significant (*p* < 0.05). Overall, the AUC values of the above 13 indicators were greater than 0.7 (Table 2 and Figure 3), indicating that they have predictive value for ADLs in middle-aged and elderly people in China.

**Table 2:** Cut-off between area under the curve for obesity and lipid-related indices to detect ADL by sex.

	Male				Female			
	AUC	AUC 95% CI	Optimal cutoffs	*P*_Value	AUC	AUC 95% CI	Optimal cutoffs	*P*_Value
BMI	0.773	0.658–0.887	25.748	<0.001	0.721	0.678–0.765	26.142	<0.001
WC	0.748	0.634–0.863	89.950	<0.001	0.713	0.667–0.759	88.950	<0.001
WHtR	0.737	0.617–0.858	0.551	<0.001	0.717	0.672–0.762	0.587	<0.001
CVAI	0.758	0.652–0.864	132.354	<0.001	0.719	0.674–0.763	113.809	<0.001
VAI	0.768	0.666–0.870	3.909	<0.001	0.705	0.705–0.752	5.643	<0.001
ABSI	0.714	0.586–0.843	0.829	0.001	0.702	0.655–0.750	0.859	<0.001
BRI	0.761	0.649–0.872	4.378	<0.001	0.717	0.672–0.763	5.145	<0.001
LAP	0.793	0.704–0.882	35.669	<0.001	0.708	0.662–0.755	49.068	<0.001
CI	0.712	0.582–0.842	7.563	0.001	0.705	0.658–0.752	7.581	<0.001
TYG	0.776	0.686–0.866	8.770	<0.001	0.704	0.656–0.751	8.690	<0.001
TYG_BMI	0.790	0.692–0.887	239.694	<0.001	0.720	0.676–0.764	224.475	<0.001
TYG_WC	0.769	0.677–0.860	8.028	<0.001	0.712	0.666–0.758	8.029	<0.001
TYG_WHtR	0.737	0.617–0.858	4.891	<0.001	0.715	0.669–0.761	5.204	<0.001

WC, waist circumference; BMI, body mass index; WHtR, waist to height ratio; VAI, visceral adiposity index; ABSI, A body shape index; BRI, body roundness index; LAP, lipid accumulation product; CVAI, Chinese visceral adiposity index; CI, conicity index; TyG index, triglyceride-glucose index; TyG-BMI, TyG related to BMI; TyG-WC, TyG related to WC; TyG-WHtR, TyG related to WHtR.

Table 3 shows the odds ratios (ORs) and 95% confidence intervals (CIs) for the associations between obesity and lipid-related indices and ADL disability. The 13 obesity and lipid-related indices were transformed into two types of variables for this survey on the basis of the optimal cutoffs in Table 2. Table 3 is based on the transformed variables. In general, a larger OR indicates a greater risk factor. After adjusting for age, education, marital status, and current residence (Model 2), the odds of risk for ADL disability increased progressively with increasing units of the obesity- and lipid-related indices for both males and females. After adjusting for all covariates (Model 3), for example, in the male population, each unit increase in the LAP index was associated with a 6.62-fold (95% CI: 1.75–25.09) increase in the odds of ADL disability in men, and in the female population, each unit increase in the BMI index was associated with a 2.92-fold (95% CI: 1.94–4.40) increase in women. After adjusting for confounders, all indices were statistically significant (*p* < 0.05).

**Table 3:** Multivariate weighted logistic regression model showing the relationship between obesity and lipid-related and ADL.

Variables	Male						Female					
	Nonadjusted model		Model 1		Model 2		Nonadjusted model		Model 1		Model 2	
	OR (95% CI)	*p*	OR (95% CI)	*p*	OR (95% CI)	*p*	OR (95% CI)	*p*	OR (95% CI)	*p*	OR (95% CI)	*p*
BMI	3.85 (1.36–10.95)	0.011	6.33 (2.08–19.24)	0.001	5.02 (1.55–16.21)	0.007	2.14 (1.49–3.10)	<0.001	2.76 (1.88–4.03)	<0.001	2.92 (1.94–4.40)	<0.001
WC	3.04 (1.03–8.97)	0.044	4.31 (1.40–13.26)	0.011	3.88 (1.17–12.84)	0.027	1.79 (1.24–2.59)	0.002	1.86 (1.28–2.70)	0.001	1.95 (1.30–2.94)	0.001
WHtR	4.50 (1.42–14.25)	0.011	5.74 (1.76–18.70)	0.004	3.93 (1.15–13.43)	0.029	2.27 (1.57–3.30)	<0.001	2.03 (1.39–2.96)	<0.001	1.99 (1.33–2.98)	<0.001
CVAI	2.03 (0.71–5.77)	0.185	2.59 (0.88–7.63)	0.084	1.94 (0.63–5.93)	0.246	2.73 (1.85–4.04)	<0.001	2.08 (1.38–3.14)	<0.001	2.22 (1.43–3.45)	<0.001
VAI	3.11 (1.10–8.83)	0.033	4.60 (1.53–13.80)	0.006	3.71 (1.17–11.72)	0.026	1.70 (1.17–2.45)	0.005	1.74 (1.20–2.53)	0.003	1.62 (1.09–2.42)	0.017
ABSI	1.78 (0.63–5.05)	0.278	1.40 (0.47–4.18)	0.542	0.90 (0.28–2.90)	0.857	2.14 (1.48–3.10)	<0.001	1.47 (1.00–2.18)	0.051	1.27 (0.85–1.89)	0.250
BRI	4.50 (1.42–14.25)	0.011	5.74 (1.76–18.70)	0.004	3.93 (1.15–13.43)	0.029	2.27 (1.57–3.30)	<0.001	2.03 (1.39–2.96)	<0.001	1.99 (1.33–2.98)	<0.001
LAP	4.33 (1.37–13.73)	0.013	6.76 (2.00–22.84)	0.002	6.62 (1.75–25.09)	0.005	1.82 (1.26–2.63)	0.001	1.91 (1.32–2.78)	<0.001	1.77 (1.18–2.66)	0.006
CI	2.91 (1.02–8.25)	0.045	2.59 (0.88–7.60)	0.083	2.04 (0.68–6.15)	0.206	1.85 (1.28–2.67)	0.001	1.39 (0.95–2.04)	0.090	1.25 (0.84–1.86)	0.275
TYG	3.34 (1.13–9.87)	0.029	5.18 (1.66–16.16)	0.005	3.44 (1.06–11.13)	0.039	1.50 (1.03–2.18)	0.034	1.52 (1.04–2.22)	0.032	1.40 (0.92–2.12)	0.117
TYG_BMI	5.61 (1.97–15.97)	0.001	9.91 (3.19–30.79)	<0.001	8.57 (2.47–29.69)	<0.001	2.15 (1.48–3.12)	<0.001	2.58 (1.76–3.78)	<0.001	2.94 (1.93–4.50)	<0.001
TYG_WC	2.81 (0.99–7.98)	0.052	4.36 (1.46–12.98)	0.008	3.88 (1.17–12.84)	0.027	1.99 (1.38–2.88)	<0.001	2.04 (1.40–2.96)	<0.001	2.00 (1.31–3.05)	0.001
TYG_WHtR	2.17 (0.78–6.03)	0.140	3.21 (1.10–9.33)	0.033	2.21 (0.72–6.75)	0.165	2.08 (1.44–3.01)	<0.001	1.93 (1.33–2.81)	<0.001	1.76 (1.16–2.66)	0.007

Unadjusted model, not adjusted for any variables; Model 1, adjusted for age, marital, education and residence; Model 2, male, adjusted for age, sleephr_lunch, frailty, hypertension; female, adjusted for age, marital, residence, life_satisfy, hypertension, diabetes, frailty, height, Sleephr_night.

## 4 Discussion

On the basis of a large sample of CHARLS datasets, this study systematically evaluated the relationships between 13 obesity and lipid-related indicators and ADL disability in Chinese middle-aged and elderly individuals and compared the predictive efficacy of these indicators for the risk of ADL disability. The results of the present study revealed that, after basic population information such as age, education, and marital status was combined, all the indicators were significant predictors of ADL disability in Chinese middle-aged and elderly people. After adjusting for confounders, the odds of developing ADL disability increased gradually with increasing obesity and lipid-related index units. We also found that the LAP index was the best predictor of ADL disability in men (AUC = 0.793), followed by the TYG-BMI (AUC = 0.790), and that BMI was the most accurate predictor in women (AUC = 0.721), followed by the TYG-BMI (AUC = 0.720), according to the ROC analysis.

ADL disability refers to an individual’s inability to perform basic activities of daily living independently, requiring assistance from others in at least one activity (21). Our study revealed that the risk of ADL disability was positively associated with increased obesity and lipid-related indices in patients, which is consistent with previous findings. A longitudinal study of community-dwelling older adults suggested that underweight or obese individuals are more likely to have ADL disability than normal-weight individuals are (22). In another study of individuals aged 50–90 years, the risk of ADL disability was greater in the obese group than in the normal-weight group at any age and became more prevalent with age in obese individuals (23).

Obesity and dyslipidemia are thought to be linked to ADL disability through multiple pathways, the most important of which are thought to be obesity-related chronic diseases, including type 2 diabetes (T2D), coronary heart disease, MS, osteoarthritis of weight-bearing joints, multiple cancers, and cardiovascular disease (CVD) (24, 25). Adipose tissue in obese individuals (especially those with visceral adiposity) may play an active endocrine role in the secretion of proinflammatory cytokines (e.g., TNF-α, IL-6, and MCP-1) and adipokines, leading to the activation of chronic low-grade inflammation. Chronic low-grade inflammation has been shown to be associated with the onset and progression of several chronic noncommunicable diseases, with the strongest empirical evidence for MS, T2D, and CVD, which ultimately increase the risk of ADL disability (26–28). On the other hand, obesity may be associated with frailty syndrome, which may further contribute to the development of ADL disability (29).

Notably, the present study revealed that the LAP index was the best predictor of ADL disability in men compared with BMI in women. LAP is an index used to assess lipid accumulation, particularly that of central lipids. It is calculated from waist circumference (as an indicator of visceral fat content) and fasting circulating triglyceride levels, has the ability to represent lipotoxicity, has been suggested to be a better indicator of MS and CVD risk, and is superior to BMI in identifying T2D risk (30–32). Considering the limitations of BMI in distinguishing between muscle mass and fat mass-related body weight and that men have higher muscle mass and more muscular individuals, there may be limitations in the ability of BMI to predict ADL risk in men (17). In contrast, the LAP index may be more sensitive to abdominal fat accumulation and dyslipidemia in men; thus, the LAP index has a better predictive effect in men. In women, BMI is a relatively simple indicator of body fat distribution and differs from that in men, and BMI may be more reflective of the impact of overall obesity on body function in women. In addition, there are significant differences in the immune systems of adult males and females, which characterize chronic inflammation differently in males and females (33, 34). Previous studies have shown that BMI is an independent risk factor for chronic inflammation and that increased body mass index is more proinflammatory in women than in men; the proinflammatory effect of increased BMI is greater in women when BMI is greater than 33, whereas there is no tendency for chronic inflammation to be exacerbated in men (35). Considering the contribution of chronic inflammation to obesity-related chronic diseases and ultimately to the development of ADL disability, BMI can be considered a more sensitive predictor for women.

Our study also revealed that the TyG-BMI had a better predictive effect on both men and women. TyG-BMI, an index consisting of three easily measurable biochemical parameters (BMI, TG, and fasting glucose), is a readily available and clinically meaningful indicator of IR (36). In addition, it has also been found to have excellent diagnostic value in assessing the risk of various metabolic diseases, such as MS, prediabetes and diabetes, in recent epidemiologic investigations (37). Studies have shown that IR can affect somatic function by promoting disturbances in muscle glycolipid metabolism and impairing mitochondrial function, leading to muscle attenuation, strength loss, and neurodegeneration (38, 39). Furthermore, high TYG-BMI levels are associated with an elevated risk of coronary atherosclerosis via mechanisms that may involve increased release of chronic inflammatory factors (e.g., IL-6, TNF-α) and oxidative stress-mediated endothelial dysfunction, which in turn accelerates vascular sclerosis and limits exercise capacity (40, 41). More studies are needed in the future to further investigate the specific mechanisms, molecular pathways, and sex-specific modifiers of TYG-BMI affecting ADL disability.

### 4.1 Limitations and strengths

Our study has several limitations. Considering that the present study was a cross-sectional study, although the effect of confounding factors was reduced by multivariate adjustment. First, data of this study included only Chinese people aged 45 years or older, and the generalizability of these findings to other countries and ethnic groups may need to be further explored. Second, long-term follow-up data to assess the effects of obesity and lipid-related indicators on ADL disability over time are lacking. Future studies need to further validate the predictive ability of these indicators through longitudinal designs.

Our study has several strengths. First, we analyzed the effects of 13 obesity- and lipid-related indicators on ADL disability, comprehensively compared their ability to predict ADL disability, and identified the optimal cutoff point for each indicator through receiver operating characteristic (ROC) analysis, which can aid early screening and intervention. Second, most of the indicators in this study are simple indicators with strong utility, and these data are easier to obtain and can be widely used in clinical practice. In addition, the study analyzed males and females separately, revealing sex differences in the prediction of ADL disability and providing a basis for individualized intervention.

## 5 Conclusion

In this study, we found that all 13 obesity indices and lipid-related indices were associated with ADL disability and significantly predicted the risk of ADL disability in middle-aged and elderly Chinese individuals. Among the 13 indices, the LAP index was the best predictor of ADL disability in men, and BMI was the best predictor in women. Moreover, the odds of developing ADL disability increased with increasing obesity and lipid-related indices. Notably, these metrics can be used as simple predictors of ADL disability in clinical practice and epidemiological studies, assisting in the early identification and prevention of ADL disability.

## Data Availability

The raw data supporting the conclusions of this article will be made available by the authors, without undue reservation.
